# Predictors of Short-Term Outcomes after Syncope: A Systematic Review and Meta-Analysis

**DOI:** 10.5811/westjem.2018.2.37100

**Published:** 2018-03-13

**Authors:** Thomas A. Gibson, Robert E. Weiss, Benjamin C. Sun

**Affiliations:** * University of California, Los Angeles, Department of Biostatistics, Los Angeles, California; † Oregon Heath & Science University, Center for Policy and Research in Emergency Medicine, Department of Emergency Medicine, Portland, Oregon

## Abstract

**Introduction:**

We performed a systematic review and meta-analysis to identify predictors of serious clinical outcomes after an acute-care evaluation for syncope.

**Methods:**

We identified studies that assessed for predictors of short-term (≤30 days) serious clinical events after an emergency department (ED) visit for syncope. We performed a MEDLINE search (January 1, 1990 – July 1, 2017) and reviewed reference lists of retrieved articles. The primary outcome was the occurrence of a serious clinical event (composite of mortality, arrhythmia, ischemic or structural heart disease, major bleed, or neurovascular event) within 30 days. We estimated the sensitivity, specificity, and likelihood ratio of findings for the primary outcome. We created summary estimates of association on a variable-by-variable basis using a Bayesian random-effects model.

**Results:**

We reviewed 2,773 unique articles; 17 met inclusion criteria. The clinical findings most predictive of a short-term, serious event were the following: 1) An elevated blood urea nitrogen level (positive likelihood ratio [LR+]: 2.86, 95% confidence interval [CI] [1.15, 5.42]); 2); history of congestive heart failure (LR+: 2.65, 95%CI [1.69, 3.91]); 3) initial low blood pressure in the ED (LR+: 2.62, 95%CI [1.12, 4.9]); 4) history of arrhythmia (LR+: 2.32, 95%CI [1.31, 3.62]); and 5) an abnormal troponin value (LR+: 2.49, 95%CI [1.36, 4.1]). Younger age was associated with lower risk (LR−: 0.44, 95%CI [0.25, 0.68]). An abnormal electrocardiogram was mildly predictive of increased risk (LR+ 1.79, 95%CI [1.14, 2.63]).

**Conclusion:**

We identified specific risk factors that may aid clinical judgment and that should be considered in the development of future risk-prediction tools for serious clinical events after an ED visit for syncope.

## INTRODUCTION

### Background

There are over 1.3 million annual events of syncope (transient loss of consciousness with rapid and spontaneous recovery^1^) in the United States that lead to an emergency department (ED) visit, resulting in 440,000 admissions^2^ and $2.4 billion in yearly hospital costs.^3^ Syncope may be a harbinger of sudden death, dangerous arrhythmias, or other serious medical conditions (e.g., pulmonary embolism). Evaluation of syncope is challenging as symptoms have resolved by the time patients seek medical evaluation, and fewer than 10% of ED evaluations reveal a serious condition that may explain the episode of syncope.^4^ To mitigate the risk of sudden death or other dangerous clinical events, up to 85%^5^ of older adults who present with syncope of unclear cause are hospitalized for a diagnostic evaluation.^6^,^7^ However, admission is associated with low diagnostic and therapeutic yield,^8^,^9^ and there is no evidence that current practice patterns improve quality of life or long-term survival.^10^

According to the 2017 American College of Cardiology (ACC) / American Heart Association (AHA) / Heart Rhythm Society (HRS) Syncope Guidelines, accurate risk prediction using the clinical examination (including history taking, physical exam, and a 12-lead electrocardiogram [ECG]) is fundamental to guiding diagnostic and disposition decisions.^1^ For ED decision-making, the focus is on predicting the risk of short- term (≤30 days) events that may warrant immediate hospitalization and testing.^11^,^12^ Unfortunately, unstructured physician judgment appears to have poor reliability and accuracy.^13^

### Importance

Multiple research groups have proposed objective risk-stratification scores, each with different combinations of predictors.^4^, ^14^–^21^ However, meta-analyses of published tools suggest equivalent performance compared to unstructured provider judgment.^22^, ^23^ The 2017 ACC/ AHA/ HRS Syncope Guidelines^1^ identified several limitations of the existing literature, including small sample sizes that limit the reliability of prediction models.^24^ Although the 2017 Guidelines did identify potential predictors of short- and long-term clinical adverse events, diagnostic test characteristics of such factors were not described. Thus, it may not be apparent to front-line clinicians how to weight the presence or absence of high-risk factors identified in the clinical examination. In addition, prior attempts to develop risk scores have not incorporated existing information about potential predictors. A Bayesian approach that includes prior risk-stratification data may potentially result in the development of more reliable and valid risk scores.

### Goal of this Investigation

To address this evidence gap, we performed a systematic review and meta-analysis to describe the diagnostic test characteristics of initial history, physical exam, ECG, and selected tests.

## METHODS

### Study Design

We performed a systematic literature review and meta-analysis that complied with the Preferred Reporting Items for Systematic reviews and Meta-Analyses (PRISMA) guidelines.^25^ This study was exempt from institutional review board review.

### Search Strategy and Study Selection

We conducted English-language searches in MEDLINE using a combination of the terms “syncope” and “risk” in any field. We considered papers published between January 1, 1990, and July 1, 2017. We also manually reviewed the reference lists of relevant studies. Titles and abstracts for all articles were screened by one of the authors (BCS). The full text of potentially eligible studies was reviewed by the entire study team.

Inclusion criteria included the following: 1) Patients presenting to an ED after a syncope event; 2) serious, short-term (≤30 days) clinical outcomes (such as death, arrhythmia, structural/ ischemic cardiac events, neurovascular events, major bleeding) were reported^12^; and 3) data sufficient for potential predictors stratified by the presence of adverse outcomes. For univariate data, this meant either (a) counts of the number of patients with a given characteristic (e.g., abnormal ECG) that did have an adverse event and the number of patients with the characteristic that did not have adverse events, along with the total number of patients with and without adverse events (i.e., contingency table counts for adverse outcomes by the presence of the characteristic), or (b) odds ratios and confidence intervals. For multivariate data, we required output from a multiple logistic regression model in (a) coefficient estimates and standard errors, or (b) odd ratios (OR) and confidence intervals (CI). All the papers that we included intended to either identify predictors of adverse events after syncope or validate existing risk-stratification methods.

**Population Health Research Capsule**

What do we already know about this issue?

The emergency department evaluation relies on careful history, examination, electrocardiogram, and selective testing to evaluate patients presenting with syncope.

What was the research question?

What are the predictors of serious clinical outcomes after an acute-care evaluation for syncope?

What was the major finding of the study?

The strongest predictors included age, cardiac co-morbidities, hypotension, and abnormal biomarkers.

How does this improve population health?

We identified specific risk factors that may aid in clinical evaluation and risk prediction for patients undergoing ED evaluation for syncope.

We excluded studies that included non-ED referral populations (e.g., arrhythmia clinic, falls and syncope unit) or that were conditioned on prior testing (e.g. electrophysiology or tilt-table test) or pre-existing co-morbidities (e.g., prior cardiac arrest, cardiomyopathy). Because the focus of this review was to aid short-term decision-making relevant in the ED, we excluded studies that only had data on long-term (>30 days) outcomes. We excluded studies that had implausible results (defined as OR >20).

For each included article, we evaluated for potential bias with a modified version of the Quality Assessment of Diagnostic Accuracy Studies (QUADAS) Criteria,^26^ a meta-analytic system devised specifically for diagnostic studies. Two members of the study team (TAG, BCS) independently reviewed the methodological quality of included studies, and disagreements were settled through consensus.

### Information Extraction

Papers provided varying degrees of covariate information, and the method of extraction varied with the type of information: direct, for papers that provided exact counts for contingency tables; direct after rounding, for papers that provided percentages instead of counts; and extrapolated, for papers that provided ORs and 95% CIs (see Appendix A for detailed description of methodology). We analyzed all variables for which at least two papers provided information.

### Statistical Analysis

We calculated sensitivity, specificity, and likelihood ratio positive (LR+) and negative (LR−) for predictor variables. We did not assess diagnostic performance of specific risk scores, as two meta-analyses have previously addressed this issue.^22^,^23^ A Bayesian random effects model was used for meta-analyses. The Bayesian approach allows for direct probability statements to be made about quantities of interest; for example, the probability that the positive likelihood ratio for a given variable is above some diagnostic threshold. Additionally, all parameter uncertainty is accounted for automatically in each analysis (see Appendix A for detailed description). Positive and negative likelihood ratios were deemed statistically significant if the 95% posterior interval did not contain 1.0. All analyses were implemented in the R statistical package.^27^

We numerically assessed the level of heterogeneity of effect sizes via the mean posterior standard deviation of random effects on the log-odds ratio scale. Smaller (larger) values indicate less (more) heterogeneity. Values very close to zero would suggest a fixed-effects model (i.e., no variation in the effect size across papers), while values above 2.5 would signal a problem with the data or the model. We visually assessed heterogeneity with L’Abbé plots.^28^ The L’Abbé plot shows the event rate for those with a given covariate against those without the covariate for each paper that provided exact count data, where each circle represents a paper. Circle sizes are proportional to the square root of the sample size. Points above the line represent a higher event rate for those with the covariate than those without, and an OR or LR greater than one. Points clustered near the line suggest little or no covariate effect.

## RESULTS

Initial Medline and manual screening identified 2,773 potentially eligible studies, and we identified seven additional studies through citation reviews. Seventeen articles met our inclusion criteria and were included in the systematic review (Appendix B [eTable 1]; Appendix C [eFigure 1]).^4^,^15^,^16^,^18^,^20^,^21^,^29^–^39^ Definitions for serious clinical outcomes, abnormal ECG findings, and binary thresholds for continuous variables (such as age, vital signs, and biomarker tests) varied across papers; these are summarized in Appendix B [eTables 1–3]. Risk for bias is described in Appendix B [eTable 4]. We identified 32 predictors for which there were sufficient data for analysis. Visual representation of effect size heterogeneity is presented in Appendix C [eFigure 2]. There were 12 predictors with a significant LR+, and 12 predictors with a significant LR−. In this section, we highlight findings of LR+>2.0 and LR−<0.5 as the strongest predictors of short-term outcomes identified in this meta-analysis.

### Pretest Probability of Serious Outcomes

Rates of serious outcomes after an ED evaluation syncope ranged from 1.2–36.2% (interquartile range 6.7–16.9%). There was heterogeneity in the definition of serious outcomes across studies (Appendix B [eTable 1]) and whether patients with serious outcomes identified during the index ED visit were excluded from analysis (Appendix B [eTable 4]).

### Patient Characteristics and Co-morbidities (Table 1)

Pre-existing co-morbidities predictive of short-term outcomes included a history of congestive heart failure (LR+ 2.65, 95%CI [1.69, 3.91]) and prior arrhythmias (LR+ 2.32, 95%CI [1.31.–3.62]). Younger age was variably defined (e.g., less than 58–75; see Appendix B [eTable 2]) and was associated with lower risk of outcomes (LR− 0.44, 95%CI [0.25, 0.68]). Other patient characteristics, including gender, race and prior history of syncope were weakly predictive of short-term outcomes.

### Symptoms (Table 2)

A complaint of dyspnea was predictive (LR+ 2.29, 95%CI [1.31, 3.65]). Other symptoms, including traumatic injury after syncope, palpitations, position, effort, chest pain, and absence of prodromes, had non-significant LR+ and LR−.

### Physical Exam (Table 3)

Hypotension was variably defined (e.g., systolic blood pressure less than 80–90; see Appendix B [eTable 2]) but identified as a strong predictor (LR+2.62, 95%CI [1.12, 4.90]). Presence of cardiac murmur, rapid respiratory rate, and low oxygen-saturation level were not predictive.

### Tests (Table 4)

We identified multiple biomarkers predictive of short-term risk; thresholds used to dichotomize test values are presented in Appendix B [eTable 2]. Predictors of increased risk include elevated blood urea nitrogen (LR+ 2.86, 95%CI [1.15, 5.42]), troponin (LR+ 2.49, 95%CI [1.36, 4.10]), B-type natriuretic peptide (LR+ 2.19, 95%CI [1.14, 4.00]), and hematocrit (LR+ 2.14, 95%CI [1.21, 3.43]). A low B-type natriuretic peptide level was associated with reduced risk (LR− 0.45, 95%CI [0.15, 0.91]). The presence or absence of ECG abnormalities (Appendix B [eTable 3]) was modestly predictive (LR+ 1.79, 95%CI [1.14, 2.63]; LR− 0.6, 95%CI [0.33, 0.92]).

## DISCUSSION

In this systematic review of ED patients presenting with syncope, we identified 17 articles that contained short-term prognostic information about patient characteristics, symptoms, physical exam findings, and objective testing. We identified several predictors of short-term serious outcomes. The strongest predictors of increased risk were elevated blood urea nitrogen level, a prior history of congestive heart failure, hypotension in the ED, a history of arrhythmia, and an abnormal troponin value. Younger age was the strongest predictor of lower risk. Prognostic factors had modest specificity and poor sensitivity, and likelihood ratios suggest that no single factor is sufficient to classify patients at high risk for short-term serious events. In the absence of effective risk scores, our findings can be used to supplement clinical judgment to guide management of ED patients without an obvious cause of syncope. Our results can also inform the development of more accurate and robust risk-prediction tools. For example, these data can inform “prior” estimates of association in novel risk score development using a Bayesian framework.

Higher risk patient characteristics are mostly concordant with the 2017 AHA Guidelines, including older age, male gender, and prior cardiac co-morbidities such as heart failure, arrhythmias, and coronary artery disease. We did not find an increased event rate associated with previous episodes of syncope. A prior population-based study did suggest increased 30-day mortality associated with prior syncope^40^; however, this report was excluded from our results due to inability to extract data amenable to meta-analysis.

Despite conventional clinical teaching,^41^ we found that most symptoms were poor predictors. Dyspnea was the only factor that had significant positive and negative likelihood ratios. The absence or presence of other symptoms, including palpitations, syncope while supine, chest pain, and lack of prodromes, did not significantly alter risk. It is possible that patients had poor or inaccurate recall of the circumstances associated with syncope, which would limit the discriminating value of symptoms.

Low blood pressure was the only physical exam factor predictive of risk. Although the auscultation of cardiac murmur is suggestive of structural heart disease,^42^ this finding was not predictive of short-term serious events. A potential explanation may be poor inter-rater reliability of cardiac auscultation.^16^,^43^

Initial ECG testing is a Class I recommendation in the 2017 AHA Guidelines.^1^ We found that the ECG has modest discriminating value, consistent with a prior study that reported a <3% diagnostic yield.^44^ Troponin and B-type natriuretic peptide may be objective indicators of cardiac dysfunction, and they appear to be predictive of serious short-term risk after syncope. Low hematocrit may be associated with anemia and other chronic diseases that confer risk after syncope. Finally, our meta-analysis suggests that blood urea nitrogen is associated with short-term serious events. The biological mechanism for this observation is not clear, although patients with renal dysfunction may be at higher risks for arrhythmias, bleeding, or death.^45^,^46^

Our study builds on a prior meta-analysis of predictors of adverse outcomes in syncope.^47^ D’Ascenzo identified eight variables associated with serious outcomes (OR>5) including palpitations, exertional syncope, heart disease, bleeding, supine syncope, absence of prodromes, increasing age, and trauma related to syncope. Our meta-analysis builds on this prior effort by doing the following: focusing on studies of short-term outcomes that would be relevant to emergency physicians; including recently published studies; excluding improbable results (e.g., D’Ascenzo reported an OR=65 for palpitations); analyzing a broad set of potential predictors; and reporting test characteristics that are relevant to clinicians, such as likelihood ratio, sensitivity, and specificity. Discrepancies in the reported findings between the two studies are likely due to these methodological differences.

## LIMITATIONS

First, risk prediction studies of ED patient with syncope are characterized by heterogeneity in outcomes.^24^ The majority of investigations used composite outcomes of a broad range of serious clinical conditions, which reflects clinical reality facing the ED provider – syncope can be related to a wide range of dangerous conditions that may not be apparent on initial evaluation. Although the precise outcomes definitions varied by study, there was substantial overlap in included conditions. For example, all studies included death and arrhythmia, and most studies included such conditions including acute myocardial infarction, pulmonary embolus, and significant hemorrhage.

Second, there was heterogeneity in predictor measurement. For example, binary cutoffs for continuous variables and the definition for “abnormal” ECG results differed across studies. However, we found strong consistency in direction of effect for ECG abnormalities and most continuous variables (e.g., blood pressure, serum tests). Measurement heterogeneity would likely result in a conservative bias toward a null finding.

Third, most of the studies did not exclude patients with outcomes identified during the ED evaluation. The majority of serious clinical conditions are identified during the initial ED evaluation for syncope,^4^ and failure to exclude such patients biases risk prediction toward “obvious” events. There is also the potential for incorporation bias, where a test result is used to define the outcome (e.g., an ECG that documents a clinically significant arrhythmia). We attempted to mitigate incorporation bias by excluding studies with implausibly high associations.

Finally, we performed a meta-analysis on univariate predictors of outcomes, and it is likely that these factors are not independent of each other. This limitation underscores the importance of developing risk scores that account for the correlations among potential predictors.

## CONCLUSION

We identified specific risk predictors of short-term clinical events after an ED evaluation for syncope. Individual risk factors, symptoms, exam findings, and test results in isolation are modestly predictive of risk. These findings should be used to supplement clinical judgment and inform the development of novel risk scores.

## Supplementary Information







## Figures and Tables

**Table 1:** Test characteristics for demographics and co-morbidities.

Variable	Papers	Patients	LR+ (95% CI)	LR− (95%CI)	Sensitivity (95%CI)	Specificity (95%CI)	Posterior SD
CHF	8	40279	2.65 (1.67, 3.94)	0.73 (0.54, 0.89)	0.23 (0.17, 0.29)	0.85 (0.77, 0.92)	0.740
Arrhythmia	5	39773	2.30 (1.29, 3.60)	0.72 (0.48, 0.94)	0.14 (0.10, 0.20)	0.81 (0.70, 0.90)	0.777
Heart disease	8	44074	1.82 (1.26, 2.51)	0.73 (0.54, 0.91)	0.14 (0.10, 0.19)	0.75 (0.64, 0.84)	0.716
Older age	8	28624	1.80 (1.39, 2.36)	0.44 (0.25, 0.68)	0.11 (0.08, 0.15)	0.58 (0.43, 0.72)	0.778
Pacemaker	3	38207	1.58 (0.62, 2.96)	0.89 (0.62, 1.09)	0.11 (0.06, 0.17)	0.83 (0.70, 0.92)	0.840
Diabetes	5	39773	1.38 (0.80, 2.12)	0.88 (0.64, 1.07)	0.09 (0.06, 0.13)	0.75 (0.62, 0.86)	0.736
Male gender	9	44481	1.35 (1.07, 1.70)	0.72 (0.51, 0.94)	0.09 (0.07, 0.12)	0.55 (0.40, 0.69)	0.666
Cerebrovascular	2	36000	1.34 (0.55, 2.43)	0.87 (0.47, 1.20)	0.06 (0.03, 0.10)	0.70 (0.52, 0.86)	0.843
Arrhythmic medication	2	977	1.30 (0.37, 2.84)	0.94 (0.56, 1.25)	0.16 (0.08, 0.27)	0.77 (0.60, 0.90)	0.940
Hypertension	4	39103	1.28 (0.83, 1.76)	0.77 (0.44, 1.15)	0.08 (0.05, 0.12)	0.54 (0.37, 0.69)	0.801
Seizure	2	36014	1.01 (0.27, 2.29)	1.00 (0.68, 1.25)	0.05 (0.03, 0.09)	0.79 (0.62, 0.91)	0.897
Hispanic	2	3061	0.97 (0.35, 1.94)	1.02 (0.64, 1.36)	0.08 (0.04, 0.13)	0.70 (0.51, 0.85)	0.853
Stroke	2	3268	0.81 (0.24, 1.78)	1.08 (0.73, 1.40)	0.06 (0.03, 0.10)	0.73 (0.54, 0.87)	0.886
Previous syncope	4	3908	0.73 (0.30, 1.39)	1.09 (0.88, 1.30)	0.08 (0.05, 0.12)	0.76 (0.60, 0.88)	0.807
Nonwhite Race	3	38391	0.67 (0.30, 1.18)	1.24 (0.87, 1.64)	0.04 (0.02, 0.06)	0.59 (0.41, 0.76)	0.791

*CHF*, congestive heart failure; *CI*, confidence interval, *SD*, standard deviation; Posterior SD is the SD of random effects.

**Table 2:** Test characteristics for symptoms.

Variable	Papers	Patients	LR+ (95% CI)	LR− (95%CI)	Sensitivity (95%CI)	Specificity (95%CI)	Posterior SD
Dyspnea	6	4772	2.29 (1.31, 3.65)	0.78 (0.57, 0.94)	0.25 (0.18, 0.33)	0.85 (0.75, 0.92)	0.765
Trauma	2	3254	1.68 (0.70, 3.00)	0.76 (0.38, 1.12)	0.11 (0.06, 0.18)	0.72 (0.54, 0.87)	0.850
Palpitations	4	1713	1.51 (0.59, 3.02)	0.91 (0.66, 1.09)	0.18 (0.11, 0.27)	0.84 (0.72, 0.92)	0.900
Supine	2	915	1.42 (0.55, 2.66)	0.82 (0.36, 1.29)	0.17 (0.09, 0.28)	0.66 (0.45, 0.83)	0.987
Effort	3	1420	1.36 (0.51, 2.64)	0.91 (0.59, 1.17)	0.14 (0.08, 0.22)	0.78 (0.62, 0.89)	0.887
Chest Pain	3	3561	1.18 (0.45, 2.30)	0.96 (0.66, 1.20)	0.12 (0.07, 0.19)	0.77 (0.62, 0.89)	0.886
No Prodromes	7	8980	1.10 (0.72, 1.51)	0.93 (0.65, 1.21)	0.09 (0.06, 0.13)	0.58 (0.43, 0.72)	0.810

*CI*, confidence interval, *SD*, standard deviation; Posterior SD is the SD of random effects.

**Table 3:** Test characteristics for physical findings.

Variable	Papers	Patients	LR+ (95% CI)	LR− (95%CI)	Sensitivity (95%CI)	Specificity (95%CI)	Posterior SD
Hypotension	5	4540	2.62 (1.12, 4.90)	0.82 (0.59, 0.98)	0.25 (0.17, 0.35)	0.89 (0.82, 0.95)	0.936
Respiratory Rate	2	4714	2.26 (0.83, 4.44)	0.74 (0.38, 1.04)	0.15 (0.08, 0.24)	0.81 (0.66, 0.92)	0.869
Murmur	4	3792	1.86 (0.84, 3.47)	0.83 (0.55, 1.04)	0.20 (0.13, 0.30)	0.82 (0.70, 0.91)	0.873
Oxygen	3	1660	1.49 (0.77, 2.46)	0.79 (0.45, 1.12)	0.15 (0.09, 0.22)	0.68 (0.49, 0.84)	0.807

*CI*, confidence interval; *SD*, standard deviation; Posterior SD is the SD of random effects.

**Table 4:** Test characteristics for biomarkers and electrocardiogram

Variable	Papers	Patients	LR+ (95% CI)	LR− (95%CI)	Sensitivity (95%CI)	Specificity (95%CI)	Posterior SD
Urea	2	4535	2.86 (1.15, 5.42)	0.57 (0.23, 0.96)	0.20 (0.10, 0.31)	0.79 (0.63, 0.91)	0.946
Troponin	3	6952	2.49 (1.36, 4.10)	0.54 (0.26, 0.87)	0.19 (0.11, 0.27)	0.75 (0.59, 0.87)	0.857
Creatinine	2	4535	2.43 (0.96, 4.59)	0.65 (0.29, 1.01)	0.16 (0.08, 0.26)	0.78 (0.62, 0.90)	0.913
BNP	2	663	2.19 (1.14, 4.00)	0.45 (0.15, 0.91)	0.35 (0.20, 0.50)	0.65 (0.41, 0.84)	0.877
Hematocrit	8	9354	2.14 (1.21, 3.43)	0.85 (0.67, 0.97)	0.19 (0.14, 0.26)	0.88 (0.80, 0.93)	0.799
ECG	7	5114	1.79 (1.14, 2.63)	0.60 (0.33, 0.92)	0.20 (0.13, 0.28)	0.65 (0.51, 0.78)	1.083

*BNP,* b-type natriuretic peptide; *ECG,* electrocardiogram; *CI*, confidence interval, *SD*, standard deviation; Posterior SD is the SD of random effects.
