# Supplementary material for: Predictors of Short-Term Outcomes after Syncope: A Systematic Review and Meta-Analysis
Source: West J Emerg Med. 2018 Mar 13;19(3):517–23. doi: 10.5811/westjem.2018.2.37100 (PMC5942019; doi:10.5811/westjem.2018.2.37100)
Supplement: Supplementary file 1 [file wjem-19-517-s001.docx]

**Appendix A.**

Information Extraction

We define the different approaches to information extraction, conditional on available information in each paper:

- Direct. Odds ratios and standard errors for the log-odds ratios could be calculated from exact contingency table counts.
- Direct after rounding. The paper provided percentages of patients with a given risk factor stratified by presence of an adverse event along with total sample sizes for those with events and those without. For example, a paper might provide: 30% of those with an adverse event were greater than 65 years of age, 15% of those without an adverse event were greater than 65, and there were 100 and 500 patients in each respective group. The percentages are multiplied by the total sample sizes and rounded to the nearest whole number, and these are then used as contingency table counts to calculate log-odds ratios and standard errors.
- Extrapolated. If a paper provided odds ratios and 95% confidence intervals, the log was taken of the point estimate and the endpoints of the confidence interval, and the standard error was extrapolated under the assumption that a normal approximation was used.

Analysis

A binomial sampling distribution was assumed when contingency table counts were available, and a normal prior distribution was used for the log-odds ratio with random effects for each paper included in each meta-analysis. Given posterior estimates of the log-odds ratio, we used the inverse logit function to obtain the conditional probability of an event given the presence or absence of a covariate. To calculate statistics such as sensitivity/specificity and LR+/LR- we need joint, unconditional probabilities representing the probability of landing in any of the four cells in a contingency table. Thus, we need the probability of the presence or absence of the covariate. For each paper, we assumed a binomial sampling distribution for the number of people with a covariate given the total sample size, and model the logit of the probability of having the covariate with a normal prior distribution. We then obtain posterior estimates for the probability of the presence of the covariate, and multiply the conditional probabilities of an event or no event given covariate/no covariate by the probability of having the covariate or not having the covariate to obtain the four desired joint probabilities. From here we are able to produce the posterior distribution of any statistic from a contingency table.

Implementation of the above method for obtaining joint probabilities method requires generating some number of new observations (predictions) for each of these parameters in each iteration of MCMC sampling, averaging across these predicted values, and using those averages to calculate probabilities in the method described above.
