# Supplementary material for: Predictors of Short-Term Outcomes after Syncope: A Systematic Review and Meta-Analysis
Source: West J Emerg Med. 2018 Mar 13;19(3):517–23. doi: 10.5811/westjem.2018.2.37100 (PMC5942019; doi:10.5811/westjem.2018.2.37100)
Supplement: Supplementary file 2 [file wjem-19-517-s002.docx]

**Appendix B.**

**eTable 1. Study Characteristics**

| **Paper** | **Sample Size** | **Event Rate (%)** | **Study Location** | **Extraction Type** | **Clinical Endpoint** | **Time Horizon** | **Study Type** | **Ages allowed** | **Rate of admission (%)** | **Variables** |
| --- | --- | --- | --- | --- | --- | --- | --- | --- | --- | --- |
| Costantino 2008 | 676 | 6.1 | Italy | Direct | Death, major therapeutic procedures, early readmission to hospital | 10 days | Prospective | 18+ | 32.5 | ECG, Age, Cardiovascular, No Prodromes, Male Gender, Trauma, Hypertension, Diabetes, Previous Syncope |
| Derose 2012 | 22189 | 1.2 | USA | Direct | 30-day mortality | 30 days | Retrospective | 18+ | 34.1 | Age, Heart Disease, Male Gender, Cerebrovascular, Arrhythmia, CHF, Hypertension, Diabetes, Seizure |
| Gabayan 2010 | 35330 | 2.5 | USA | Direct | cardiac death and hospitalization or procedure consistent with ischemic heart disease, valvular disease, or arrhythmia | 7 days | Retrospective | 18+ | NA | Heart Disease, Male Gender, Cerebrovascular, Arrhythmia, CHF, Hypertension, Diabetes, Race, Seizure, Pacemaker, Myocardial Infarction |
| Grossman 2007 | 293 | 23.2 | USA | Direct after rounding | Interventions: pacemaker/implantable cardiac defibrillator placement, percutaneous coronary intervention, or surgery, blood transfusion, cardiopulmonary resuscitation (CPR), alterations in antidysrhythmic therapy, endoscopy with intervention, or correction of carotid stenosis Events: death, pulmonary embolus, stroke, severe infection/sepsis, ventricular dysrhythmia, atrial dysrhythmia (including SVT [supraventricular tachycardia] and atrial fibrillation with rapid ventricular response), intracranial bleed, hemorrhage, myocardial infarction, cardiac arrest, or life-threatening sequelae of syncope (i.e., Rhabomyolysis, long bone or cervical spine fractures). | 30 days | Prospective | 18+ | 68 | ECG, Heart Disease, Male Gender, Arrhythmia, Palpitations, Effort, CHF, Dyspnea, Hematocrit, Hypotension, Previous Syncope, Chest Pain, Arrhythmic Medication, Murmur, Pacemaker |
| Isbitan 2016 | 113 | 36.2 | USA | Direct | death, acute MI, life threatening arrhythmia (ventricular fibrillation, sustained ventricular tachycardia, or asystole), a decision to implant a pacemaker/cardiac defibrillator within one month of collapse, stroke, and major bleeding requiring blood transfusion of >2 units or acute surgical or endoscopic intervention | 30 days | Prospective | 18+ | NA | BNP |
| Kayayurt 2011 | 231 | 16.9 | Turkey | Direct | Death, need for major therapeutic procedures, early readmission to hospital | 7 days | Prospective | 18+ | NA | ECG, Age, Heart Disease, No Prodromes, Palpitations, Supine, Effort, CHF, Dyspnea, Hematocrit, Murmur |
| Numeroso 2014 | 200 | 25 | Italy | Extrapolated | syncopal recurrences with severe injuries (meaning traumatic brain injury more than mild, long bones fractures, internal organ injuries); major therapeutic procedures (cardiopulmonary resuscitation; intensive care unit admission; pacemaker or implantable cardioverter-defibrillator insertion; other invasive manoeuvres, such as percutaneous coronary intervention or electrophysiology study; cardiac surgery); cardiovascular events (acute myocardial infarction; life-threatening arrhythmia; deep vein thrombosis and pulmonary embolism; cerebrovascular accident, intracranial haemorrhage, or subarachnoid haemorrhage); death for any reason | 30 days | Prospective | 18+ | NA | Male Gender |
| Numeroso 2016 | 347 | 8.4 | USA | Extrapolated | 1) death for any cause; 2) acute cardiovascular events such as myocardial infarction, malignant arrhythmias, pulmonary embolism, or stroke; 3) major acute bleeding (i.e., those requiring blood transfusion); 4) major procedures such as pacemaker (PM) or ICD implantation, electrophysiology study with radiofrequency ablation, percutaneous coronary interventions, cardiothoracic or vascular surgery, or operative gastrointestinal endoscopy; and 5) syncopal recurrences causing major trauma | 30 days | Prospective | 18+ | NA | Age, No Prodromes, Male Gender, Previous Syncope |
| Quinn 2004 | 684 | 11.5 | USA | Direct | Death, arrhythmia, myocardial infarction, pulmonary embolism, stroke, subarachnoid hemorrhage, significant hemorrhage, or any condition, causing a return ED visit and hospitalization for a related event | 7 days | Prospective | NA | 54.9 | Heart Disease, No Prodromes, Arrhythmia, Palpitations, Supine, Effort, CHF, Dyspnea, Hematocrit, Hypotension, Oxygen, Hypertension, Diabetes, Previous Syncope, Chest Pain, Seizure, Stroke, Arrhythmic Medication, Respiratory Rate, Murmur |
| Quinn 2011 | 684 | 11.5 | USA | Direct | sudden death, MI (defined as any elevation of troponin or ECG change with an accompanying diagnosis of MI on the discharge diagnosis), any arrhythmia captured on monitoring and thought to have had a temporal relationship to the syncopal event, and any structural heart disease (primarily valvular) thought to have caused the event. We also considered any acute cardiac intervention such as pacemaker insertion and cardiac catheterization as important cardiac outcomes. | 7 days | Prospective | NA | 54.9 | ECG |
| Reed 2010 | 550 | 7.5 | Scotland | Extrapolated | 1) acute myocardial infarction (MI) according to the universal definition (19); 2) lifethreatening arrhythmia (ventricular fibrillation, sustained ventricular tachycardia [ 120 beats/min], ventricular pause [ 3 s], ventricular standstill, or asystole); 3) decision to implant a pacemaker or cardiac defibrillator within 1 month of index collapse; 4) pulmonary embolus (confirmed on lung perfusion scan or CT pulmonary angiography); 5) cerebrovascular accident, intracranial hemorrhage, or subarachnoid hemorrhage (demonstrated by brain imaging or lumbar puncture); 6) hemorrhage requiring a blood transfusion of 2 U; or 7) acute surgical procedure or endoscopic intervention. Secondary end points were cardiovascular serious outcome (acute MI, arrhythmia, pacemaker/defibrillator implantation, or cardiac procedure) and syncope-related death (death due to cause of presenting syncopal episode) | 30 days | Prospective | 16+ | 48 | Male Gender, Hematocrit (Hemoglobin), Oxygen, BNP, |
| Reed 2012 | 338 | 7.4 | USA | Direct | primary endpoints were a composite at 1 month and at 1 year of serious outcomes: allcause death, acute myocardial infarction (AMI), life-threatening arrhythmia, insertion of a pacemaker or internal cardiac defibrillator device, pulmonary embolus, cerebrovascular accident or subarachnoid haemorrhage, haemorrhage requiring a blood transfusion of two units or more, or an acute surgical procedure or endoscopic intervention. Secondary endpoints were: all-cause death and serious cardiovascular outcome including cardiac death | 30 days | Prospective | 16+ | 50 | Troponin |
| Sarasin 2003 | 175 | 17.5 | Switzerland | Extrapolated | Arrhythmia | NA | Prospective | 18+ | NA | ECG, Age, CHF, Myocardial Infarction |
| Sun 2007 | 477 | 11.7 | USA | Direct after rounding | death, myocardial infarction, arrhythmias, pulmonary embolism, stroke or transient ischemic attack, subarachnoid or nontraumatic cerebral hemorrhage, aortic dissection, new diagnosis of structural heart disease thought to be related to syncope, and significant hemorrhage or anemia requiring blood transfusion | 7 days | Prospective | 18+ | 51 | ECG, Age, Male Gender, CHF, Dyspnea, Hematocrit, Hypotension, Race, Hispanic |
| Sun 2009 | 2584 | 6.7 | USA | Direct after rounding | Death, arrhythmia, myocardial infarction, new diagnosis of structural heart disease, pulmonary embolism, aortic dissection, stroke/transient ischemic attack, subarachnoid or nontraumatic cerebral hemorrage, significant hemorrhage or anemia requiring blood transfusion | 30 days | Retrospective | 60+ | 43 | ECG, Heart Disease, No Prodromes, Male Gender, Arrhythmia, Trauma, CHF, Dyspnea, Hematocrit, Hypotension, Troponin, Hypertension, Diabetes, Previous Syncope, Race, Chest Pain, Stroke, Murmur, Hispanic, Pacemaker, |
| Thiruganasambandamoorthy 2014 | 505 | 9.7 | Canada | Direct | Death, arrhythmia, myocardial infarction, diagnosis of serious underlying structural heart disease, procedural interventions to treat cause of syncope | 30 days | Retrospective | 16+ | NA | Age, Heart Disease, No Prodromes, Arrhythmia, Palpitations, Effort, CHF, Dyspnea, Hematocrit, Hypotension, Oxygen, Urea, Hypertension, Diabetes, Creatinine |
| Thiruganasambandamoorthy 2016 | 4030 | 3.6 | Canada | Direct | death, arrhythmia, myocardial infarction, serious structural heart disease, aortic dissection, pulmonary embolism, severe pulmonary hypertension, severe hemorrhage, subarachnoid hemorrhage, any other serious condition causing syncope and procedural interventions for the treatment of syncope | 30 days | Prospective | 16+ | 9.5 | Age, Heart Disease, No Prodromes, Male Gender, Hematocrit, Troponin, BNP, Respiratory Rate, Creatinine |

**eTable 2. Cutoffs for Continuous Variables**

| **Author** | **Age** | **Oxygen (%)** | **Respiratory Rate (breaths/min)** | **Urea (mmol/L)** | **Troponin (ng/ml)** | **Creatinine (mmol/L)** | **BNP (pg/ml)** | **Hematocrit (%)** | **Hemoglobin (g/l)** | **Hypotension (mm Hg)** |
| --- | --- | --- | --- | --- | --- | --- | --- | --- | --- | --- |
| Costantino 2008 | 65 |  |  |  |  |  |  |  |  |  |
| Derose 2012 | 60 |  |  |  |  |  |  |  |  |  |
| Gabayan 2010 |  |  |  |  |  |  |  |  |  |  |
| Grossman 2007 |  |  |  |  |  |  |  | 30 |  | 90 |
| Isbitan 2016 |  |  |  |  |  |  | 250 |  |  |  |
| Kayayurt 2011 | 58 |  |  |  |  |  |  | 30 |  |  |
| Numeroso 2014 |  |  |  |  |  |  |  |  |  |  |
| Numeroso 2016 | 70 |  |  |  |  |  |  |  |  |  |
| Quinn 2004 |  | 95 | 24 |  |  |  |  | 30 |  | 90 |
| Quinn 2011 |  |  |  |  |  |  |  |  |  |  |
| Reed 2010 |  | 94 |  |  |  |  | 300 |  | 90 |  |
| Reed 2012 |  |  |  |  | 0.03 |  |  |  |  |  |
| Sarasin 2003 | 65 |  |  |  |  |  |  |  |  |  |
| Sun 2007 | 60 |  |  |  |  |  |  | 30 |  | 90 |
| Sun 2009 |  |  |  |  | 0.04 |  |  | 30 |  | 90 |
| Thiruganasambandamoorthy 2014 | 75 | 90 | 20 | 15 |  | 160 |  | 30 | 100 | 80 |
| Thiruganasambandamoorthy 2016 | 75 |  |  | 12 | 99th percentile | 150 |  | 30 |  |  |

**eTable 3: Abnormal ECG definitions**

| **Author** | **Abnormal ECG Definition** |
| --- | --- |
| Costantino 2008 | atrial fibrillation or tachycardia, sinus pause greater than 2sec, sinus bradycardia with heart rate ranging b/t 35-45 bpm, conduction disorders, signs of previous myocardial infarction or ventricular hypertrophy, multiple premature ventricular beats |
| Derose 2012 | NA |
| Grossman 2007 | Abnormal heart rhythm or new ECG changes |
| Quinn 2004 | NA |
| Reed 2010 | NA |
| Sarasin 2003 | (1) atrial fibrillation; (2) sinus pause >2 and <3 seconds; (3) sinus bradycardia with heart rate of >35 beats per minute and <45 beats per minute; (4) conduction disorders (e.g., bundle branch block, second-degree Mobitz I atrioventricular block, bifascicular block); (5) signs of old myocardial infarction or ventricular hypertrophy; and (6) multiple premature ventricular beats. |
| Sun 2009 | Non-sinus rhythm, sinus rhythm with pulse rate less than 40 beats/min, Q/ST/T changes consistent with acute or chronic ischemic heart disease, abnormal conduction intervals (QRS greater than 0.1 ms, QTc greater than 450 ms), left or right ventricular hypertrophy, left axis deviation, and bundle branch block |
| Thiruganasambandamoorthy 2014 | II degree Mobitz type 2 or III degree atrioventricular block, bundle branch block and 1st degree atrioventricular block, bifascicular block, new ischemic changes, non-sinus rhythm, left axis deviation, ED cardiac monitor with sinus pauses, symptomatic arrhythmias |
| Kayayurt 2011 | Atrial fibrillation or tachycardia, sinus pause greater than 2sec, sinus bradycardia with heart rate ranging b/t 35-45 bpm, conduction disorders, signs of previous myocardial infarction or ventricular hypertrophy, multiple premature ventricular beats |
| Sun 2007 | Any rhythm other than sinus, any bundle branch block, left-axis deviation, mono- or biventricular hypertrophy, any abnormal conduction interval except for first degree atrioventricular block, any Q/ST/T change consistent with ischemia (acute or chronic), or isolated, nonspecific ST/T abnormalities |
| Gabayan 2010 | NA |
| Isbitan 2016 | NA |
| Numeroso 2014 | NA |
| Quinn 2011 | Rhythm abnormalities on the ECG (ventricular tachycardia, heart block, paced, SVT), presence of right branch bundle block (RBBB), left branch bundle block (LBBB), ST-segment changes, nonspecific ST-T wave changes, interval variants (PR, QT), presence of ectopy, and presence of Q-waves. We also reanalyzed the specific rhythm findings obtained during the separate rhythm assessment. |
| Reed 2012 | NA |
| Thiruganasambandamoorthy 2016 | NA |
| Numeroso 2016 | NA |

**eTable 4: Modified QUADAS scores**

Modified QUADAS criteria

1. Did the study prospectively enroll consecutive patients with syncope?

2. Were the patient selection criteria consistent with the 2017 AHA Syncope Guidelines (e.g. excludes patients with alternative explanations for loss of consciousness, such as seizure)?

3. Did the primary outcome includes death, arrhythmias, and/or structural/ ischemic heart disease?

4. Was the primary outcome measured within 30 days?

5. Were patients with an ED diagnosis of the primary outcome excluded from the analysis?

6. Was the procedure outcomes ascertainment described in sufficient detail to permit its replication*?*

7. Were the same clinical data available when test results were interpreted as would be available when the test is used in practice?

8. Were rates of missing data for candidate predictors reported?

9. Were patient withdrawals and attrition (e.g. lost to followup) from the study reported?

| **Reference** | **1** | **2** | **3** | **4** | **5** | **6** | **7** | **8** | **9** |
| --- | --- | --- | --- | --- | --- | --- | --- | --- | --- |
| Costantino 2008 | y | y | y | y | y | y | y | n | y |
| Derose 2012 | n | n | y | y | n | y | y | n | y |
| Gabayan 2010 | n | n | y | y | n | y | y | n | y |
| Grossman 2007 | y | y | y | y | n | y | y | n | y |
| Isbitan 2016 | y | y | y | y | n | y | y | n | y |
| Kayayurt 2012 | y | y | y | y | n | y | y | n | y |
| Numeroso 2014 | n | y | y | y | y | y | y | n | y |
| Numeroso 2016 | n | y | y | y | y | y | y | n | y |
| Quinn 2011 | y | y | y | y | n | y | y | y | y |
| Quinn 2004 | y | y | y | y | n | y | y | n | y |
| Reed 2012 | y | y | y | y | n | y | y | y | y |
| Reed 2010 | y | y | y | y | n | y | y | n | y |
| Sarasin 2003 | y | y | y | unknown | y | n | y | n | n |
| Sun 2009 | n | y | y | y | y | y | y | y | y |
| Sun 2007 | y | y | y | y | n | y | y | n | y |
| Thurgan 2016 | y | y | y | y | y | y | y | y | y |
| Thirugan 2014 | n | y | y | y | n | y | y | y | y |
