# Supplementary material for: Predictors of Short-Term Outcomes after Syncope: A Systematic Review and Meta-Analysis
Source: West J Emerg Med. 2018 Mar 13;19(3):517–23. doi: 10.5811/westjem.2018.2.37100 (PMC5942019; doi:10.5811/westjem.2018.2.37100)

**Appendix C.**

**eFigure 1.** Consort flow diagram.

Exclude (N=2720)

- Case report/review (N=339)
- No ED/acute care patients OR pediatrics only (N=275)
- No new risk prediction (e.g. validation studies of existing risk score) (n=99)
- Entirely unrelated (N=2007)

Pubmed search terms: terms in any field: ‘syncope’, 1990:2017; ‘risk’; English [language]

(N=2773)

17 studies included in meta-analysis

Exclude (N=43)

- Follow-up > 1 month (N=17)
- Improper outcome or improper data (N=17)
- Paper unavailable or data duplicate/subset of another (N=4)
- Wrong study population (N=2)
- Meta-analysis or systematic review (N=2)
- Reported odds ratio > 20 (N=1)

53 full team review

+7 identified by additional citations

(N=60)

**eFigure 2.** Heterogeneity of Effect Size Estimates.


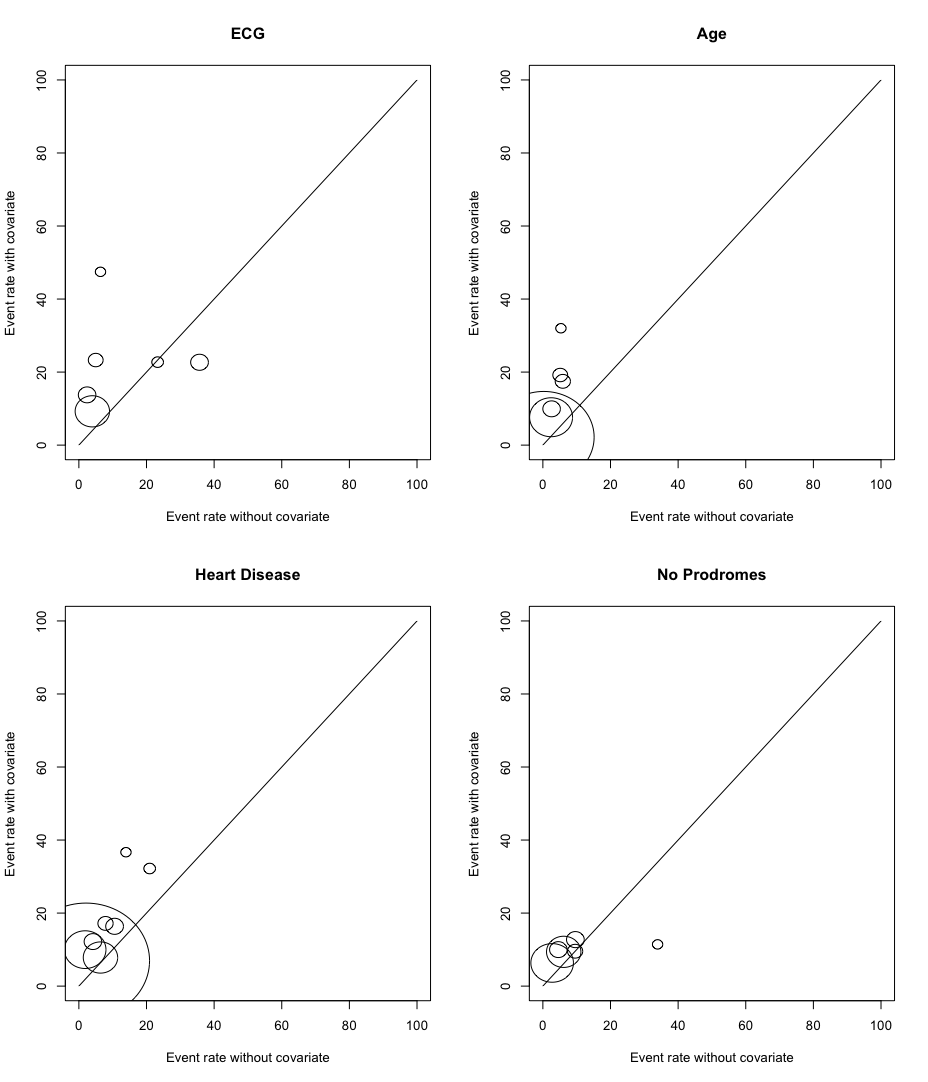


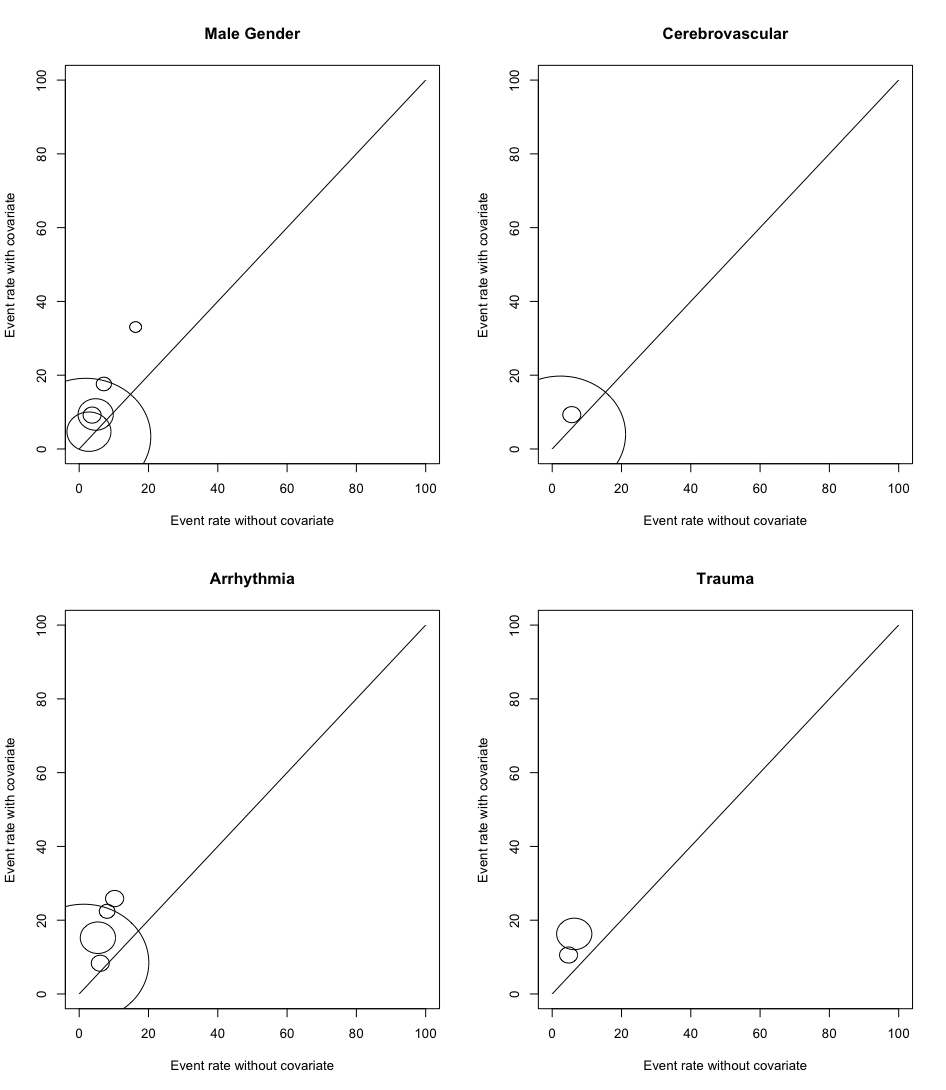


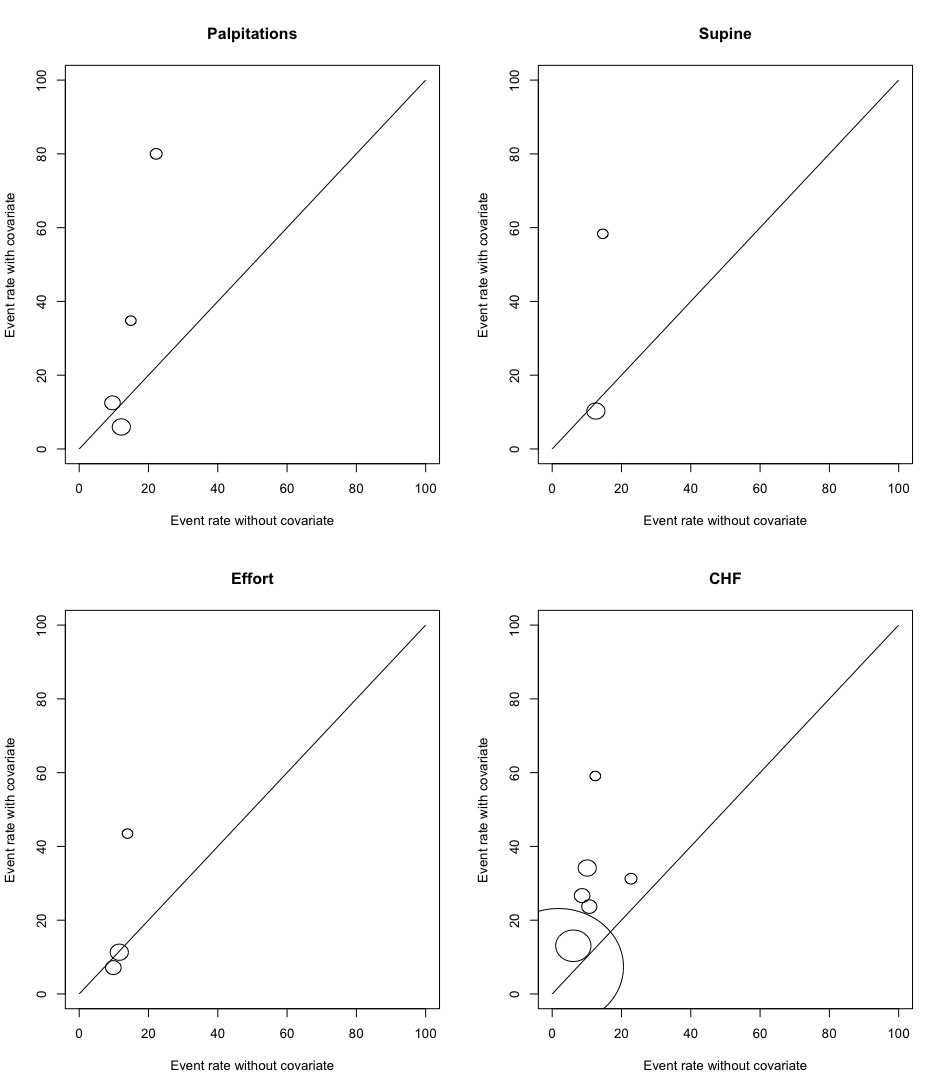


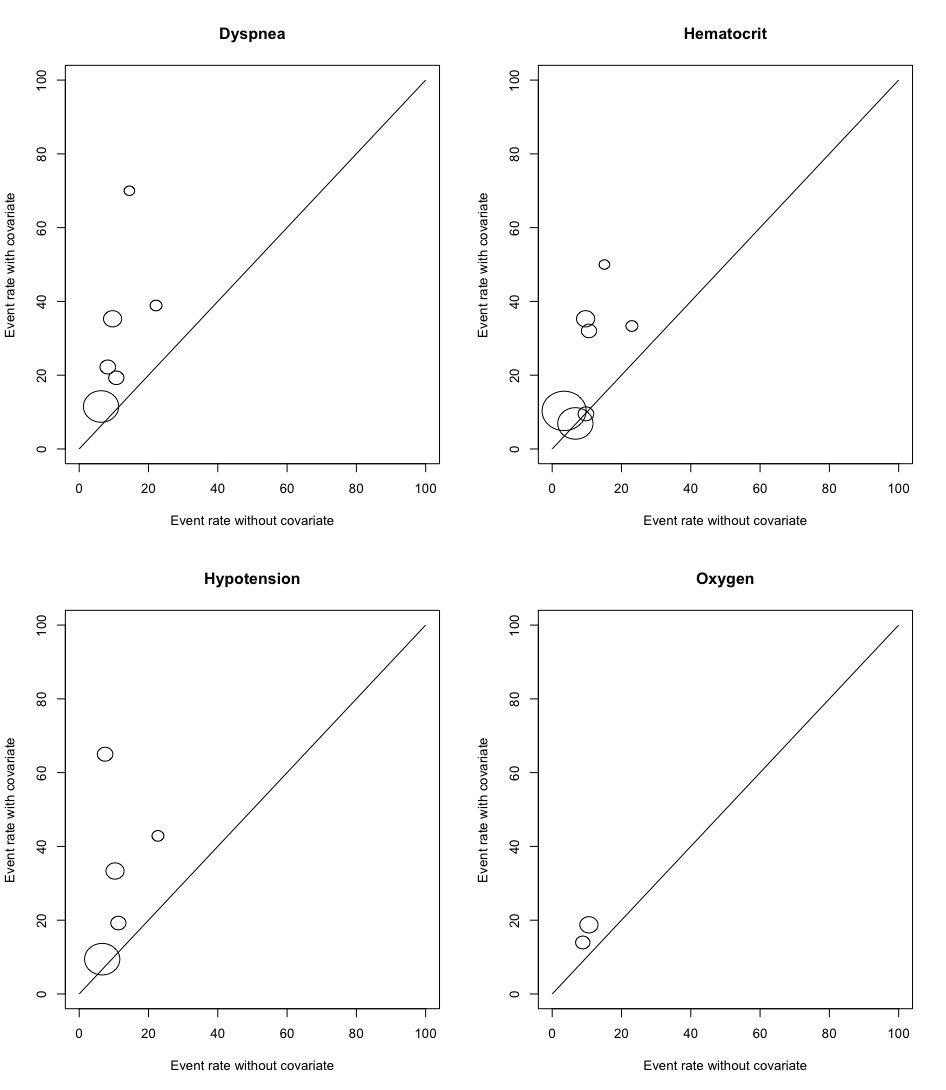


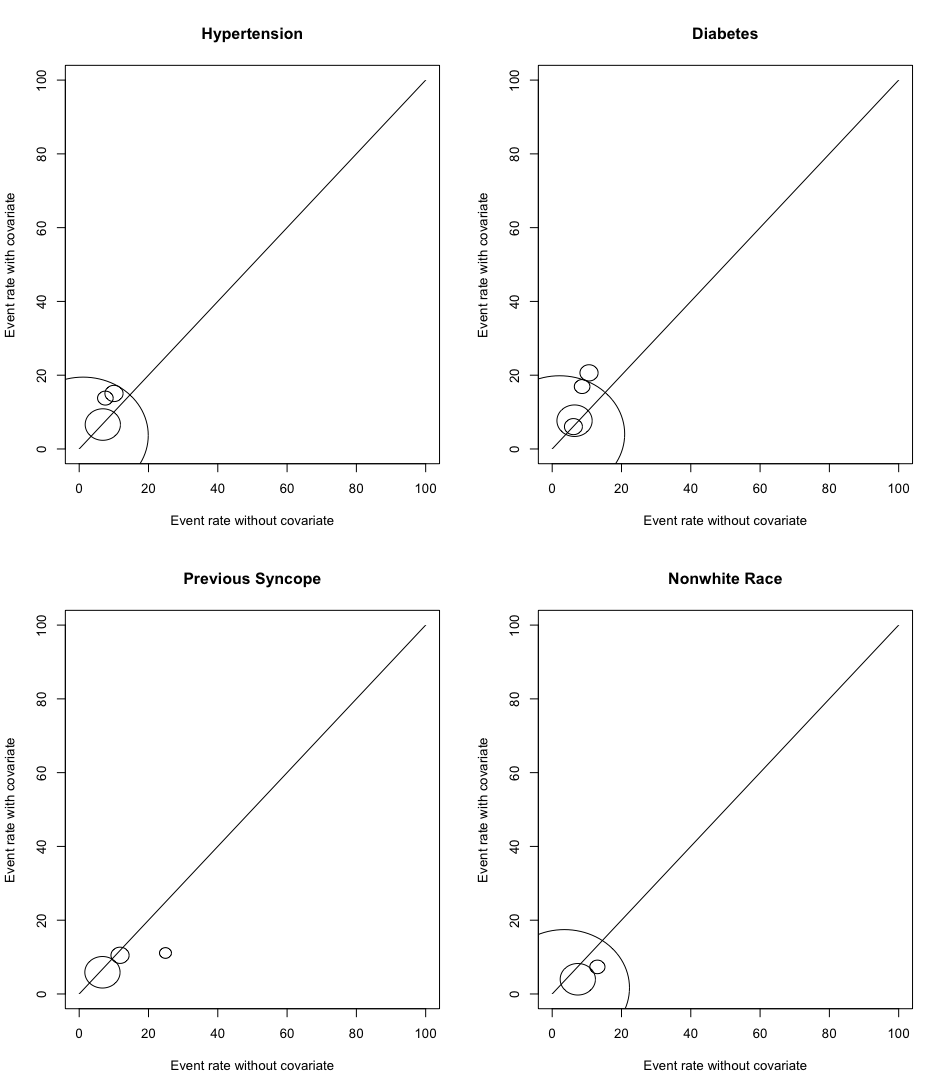


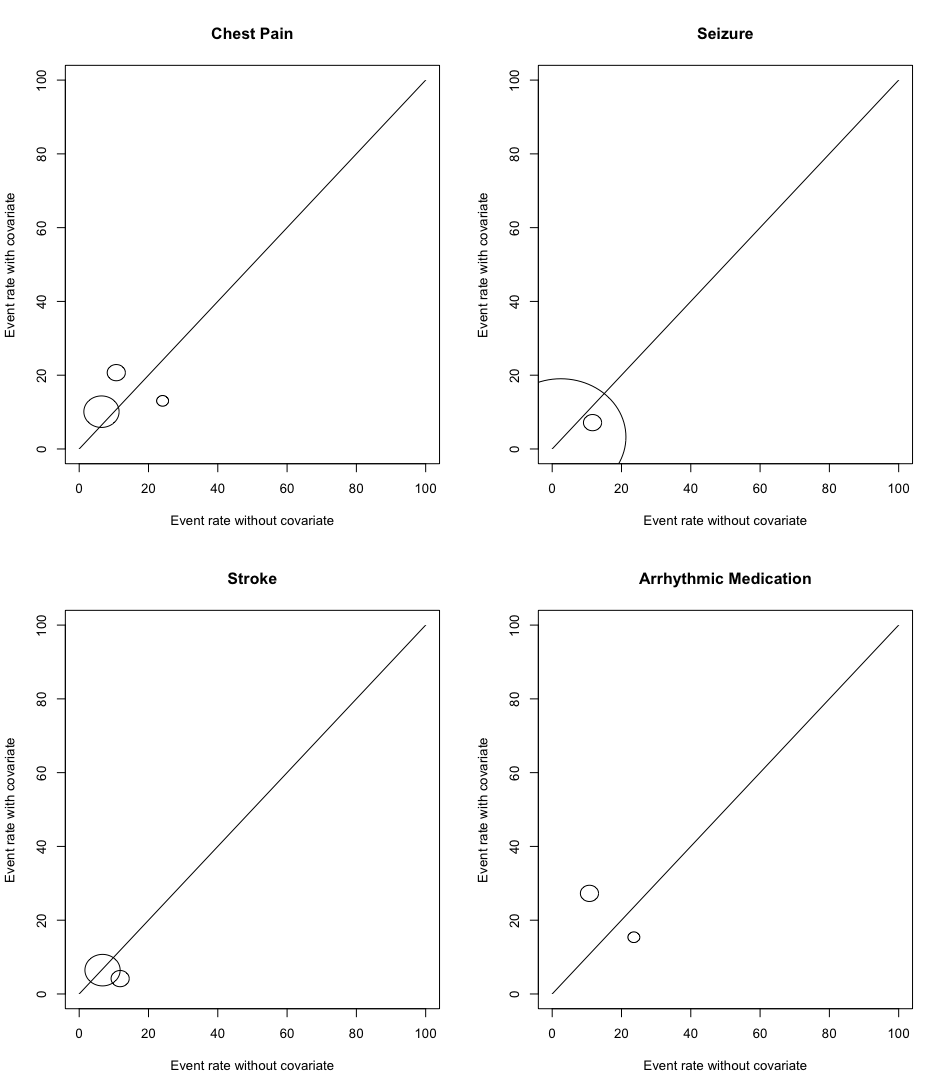


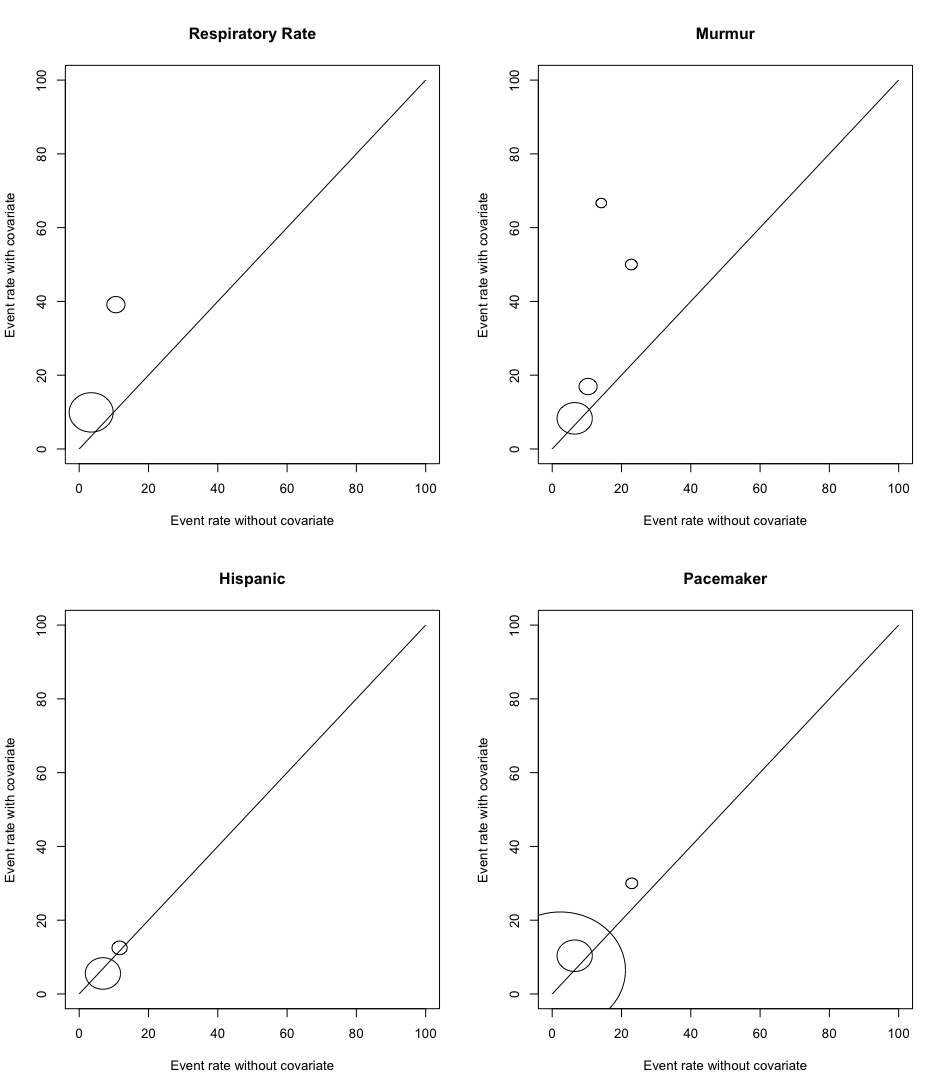


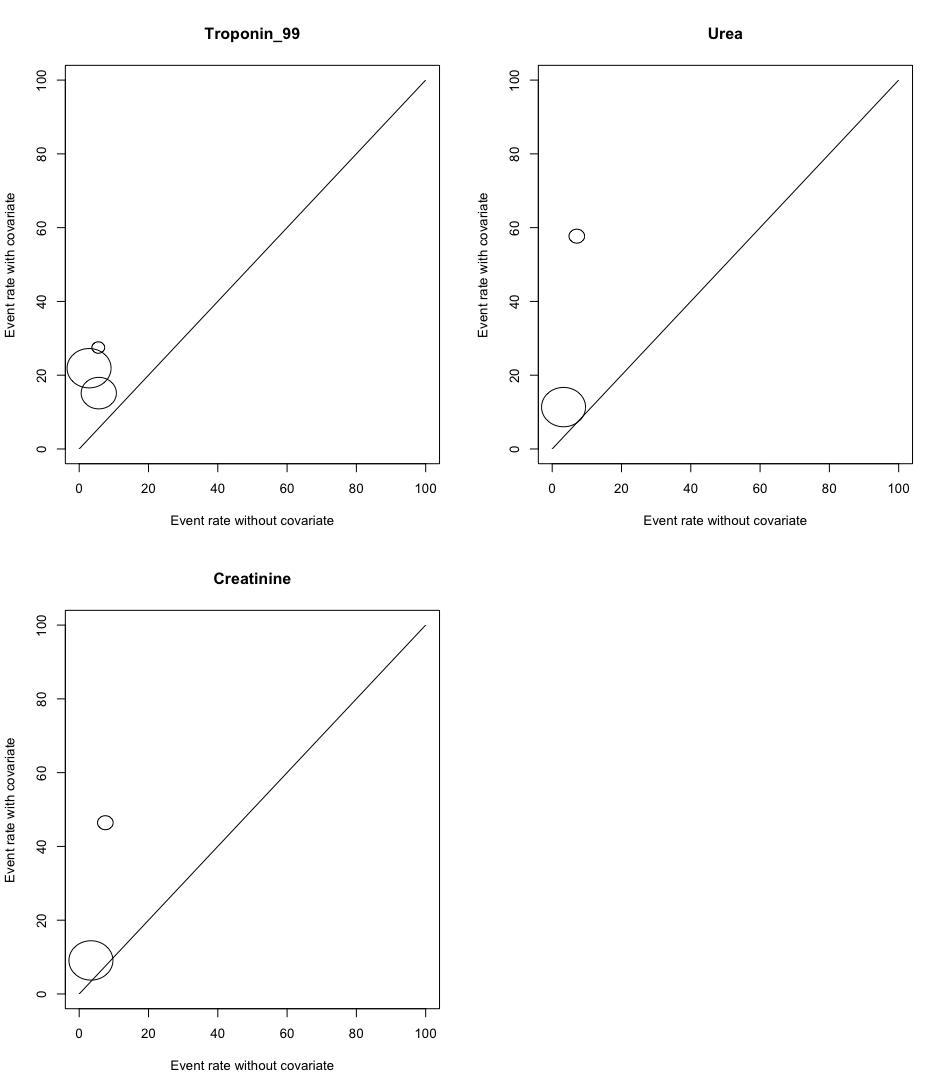

Supplement: Supplementary file 3 [file wjem-19-517-s003.docx]
